# *“Once you get cancer you die*. *There is no way to get saved from cancer*.*”* A qualitative exploration of patients’ perceptions towards cancer in Fiji

**DOI:** 10.1371/journal.pone.0277970

**Published:** 2022-12-16

**Authors:** Kaushal Kumar, Masoud Mohammadnezhad

**Affiliations:** 1 Fiji Ministry of Health and Medical Services, Lautoka Hospital, Lautoka, Fiji Islands; 2 School of Nursing and Healthcare Leadership, University of Bradford, Bradford, United Kingdom; 3 Department of Health Education and Behavioral Sciences, Faculty of Public Health, Mahidol University, Nakhon Pathom, Thailand; Public Library of Science, UNITED KINGDOM

## Abstract

**Background:**

Understanding patients’ perspective to get an insight into cancer, and how best the public health systems can battle with this disease is the way forward in this current world. This study aimed to explore patients’ knowledge about common cancers, barriers to assessing cancer information and cancer preventative approaches in Fiji.

**Methods:**

The study used a qualitative method approach that was conducted among patients who attended Special Outpatients (SOPD) at the four selected health centres in Lautoka Subdivision, Fiji from 1^st^ March to 30^th^ April 2021. A semi-structured open-ended questionnaire was used to guide in-depth interviews. These audio recordings were transcribed and analysed using thematic analysis. All interview transcripts were read and similar words and phrases were assigned numbers which were grouped together to identify themes and sub themes.

**Results:**

Twenty-eight patients took part in the in-depth interview and the responses were grouped into four themes including; cancer knowledge, diagnosis of cancer in a close friend/family, barriers of communication and optimizing cancer awareness. Patients’ awareness about common cancers and cancer risk factors was low. Many barriers for cancer screening were highlighted including stigmatization, fear, worry, death, lack of information, herbal medicine use, lack of resources and delay in diagnosis. Awareness strategies highlighted by participants included community outreach programs, house to house visits, opportunistic screening, engagement of community health care workers and the concept of a cancer hub centre.

**Conclusion:**

It is evident that there is a range of views from patients towards cancer and it is important to understand these perceptions to better guide public health interventions concerning cancer. This puts more focus on the need to invest more in information, education, and communication material for public campaigns that target a variety of people for a wider reach.

## Introduction

Cancer, is a group of illnesses that occur when abnormal body cells start to grow uncontrollably in any organ or tissue of the body and can spread to other body organs disabling their function [1–3]. Cancer is one of the main concerning Non-communicable Diseases (NCDs) around the world [4, 5]. In 2020, new reports suggested that global cancer burden has increased to 19.3 million new cases and 10 million cancer deaths worldwide. Cancers account for 29.7% of premature deaths due to NCDs globally [4, 6]. Liver, colorectal, lung, stomach and prostate cancer are the most common types of cancer in men, while lung, thyroid, cervical, breast, and colorectal cancer are the most common among women [4].

Cancer burden continues to grow rapidly across countries, putting extreme physical, emotional, and financial strain on individuals, families, communities, and health systems. By 2040, a predicted global cancer burden is expected to be more than 27 million new cancer cases per year which would almost be a 50% increase on the estimated cancer cases in 2018, with majority of the cases seen in countries with low or medium Human Development Index (HDI) [7, 8]. Many health care systems are not developed to manage this burden hence they fail to provide timely screening, diagnosis, and management of cancers [8, 9].

The importance of patient perspective in health care is being slowly recognized in modern medicine [10]. Individual perception towards an illness is influenced by many factors such as lack of knowledge about the disease and its risk factors, fear/anxiety related to the disease, barriers in accessing accurate health information and not participating in preventative health activities [11]. Majority of the population is unaware about the risk factors for cancer and are also unsure of their perceived risk to cancers [12]. Barriers have been documented in the general public and this is usually one of the reasons why people don’t access cancer screening services [13]. Public health interventions focused on changing inaccurate or unhelpful perceptions of disease is an important area in current medical practice [14]. The Leventhal’s Common Sense Model (LCSM) of illness assumes that the general public are actively processing health threat information to create common sense understandings or representations that guide health related behaviours [15–17]. Hence, it is very important to understand patients’ perspective to get an insight into cancer and how best the public health systems can battle with this disease.

With Fiji being a developing country, it is faced with many challenges in combating serious diseases. NCDs is the number one killer in Fiji with majority deaths from diabetes, hypertension, and cardiovascular diseases. Recent data published by World Health Organization (WHO) shows that Fiji recorded a total of 1487 new cancer cases in 2020 with 825 deaths [4]. To add on, patients may not be able to afford treatment for cancers since majority treatment modalities are not available locally as Fiji is still a developing country. There is a substantial amount of money being used by the government for care of cancer patients in Fiji. Much cancer costs have been attributed to cost of medications, human resources and hospital stay [18, 19]. In the National Strategic plan for Fiji Ministry of Health and Medical Services (MoHMSs), the top priority is reduction of the growing burden of NCDs including cancers [20]. With much focus on the growing numbers of diabetes and hypertension, burden of cancer in Fiji has not been studied in detail and thus it is feared that cancers are slowly creeping in and someday it could potentially become the top NCD if preventive measures are not taken. With the current global pandemic (COVID-19) in action, much of the attention has been put towards COVID-19 containment and prevention, hence cancers may move down the ladder in terms of priority.

A gap of knowledge exists since currently nil studies could be found that explores the cancer perceptions of Special Outpatients Department (SOPD) patients. Hence, this study will aim to explore patients’ knowledge about common cancers, barriers to assessing cancer information and cancer preventative approaches in Fiji.

This study will attempt to bridge the gap between the findings from western studies, and the local context and also attempt to plan targeted interventions to increase knowledge about cancers among the public.

## Methods

### Study design and setting

This study applied a qualitative approach among patients who attended SOPD in Lautoka Subdivision, Fiji from 1^st^ March to 30^th^ April 2021. The study was conducted at the four purposively selected health centres in Lautoka Subdivision namely Punjas Health Centre, Kamikamica Health Centre, Natabua Health Centre and Veiseisei Health Centre. These health centres are the busiest health centres in Lautoka Subdivision which cater for approximately 3000 patients per week and run both the General Outpatient (GOPD) services and the SOPD services for diabetes, hypertension and dyslipidaemia/cardiac. This study followed the Consolidated criteria for reporting qualitative research (S1 Checklist).

### Study sample

The study focused on all the patients who attended SOPD clinics at the four health centres in Lautoka Subdivision within the study period. The following inclusion criteria was used; SOPD patient who attended SOPD clinics at the four selected health centres in Lautoka Subdivision during the period of data collection, age more than or equal to 18 years, citizen of Fiji and lives in Lautoka (self-identified by participants) and those who agreed to participate in the study. The following exclusion criteria was used; patients not willing to participate in the study, those patients who are already diagnosed with cancer (identified through PATIS search and face to face), patients who attend SOPD clinics in other health facilities such as Lautoka Divisional Hospital and patients with mental/psychiatric conditions (since they will not be able to accurately answer the questions as they may not be in a sound state of mind).

A purposive sampling was used to select participants. Purposive sampling is a form of non-probability sampling in which the researchers rely on their own judgement when choosing members of the population to participate in their study. It is done when researchers thoroughly think through how they will establish a sample population, even if it is not statistically representative of the greater population at hand [21]. The sample size for SOPD patients was twenty-eight (28), with whom face to face in-depth interview was done until theoretical data saturation was achieved [22].

### Data collection tool

The data collection instrument used to collect data in this research was semi-structured interviews with open-ended questions. All in-depth interviews were conducted by the principal researcher himself who is by profession a medical officer. Semi-structured in-depth interviews are commonly used in qualitative research and it allows researchers to collect open-ended data, to explore participant thoughts, feelings, and beliefs about a topic and to delve deeply into personal and sometimes sensitive issues [23].

The semi structured interview questionnaire was newly developed based on the literature review and the study research questions. It had 2 sections with a total of 18 questions. The first section which was the demographics, had 7 questions followed by 11 open-ended questions in the second section. A bilingual translator was used to conduct interview with an iTaukei (native Fijian) individual.

### Study procedure

The SOPD nurses at the four health centres that is one SOPD nurse per health facility was approached and informed about the study and their help was requested in locating SOPD patients for the study but they were not involved in data collection process. Flyers containing information about the research was placed at each SOPD clinics in the four health centres at least 2 weeks prior to data collection. A short introduction of the study was also provided every week for the period of 1 month of data collection verbally by the researcher (in English and Hindi language) and one research assistant (in iTaukei language) to the patients while they waited for their turn for SOPD clinic consultation.

Together with this, verbal introduction and information sheet (English, Hindi, iTaukei) were provided to all the SOPD patients in their preferred language who fulfilled the inclusion and exclusion criteria. The patients who agreed to participate in the study at their own free time were then given consent forms (English, Hindi, iTaukei) in their preferred language. Once the participants gave their signed consents (left thumb-print if illiterate), the consent forms were collected and kept safely while the information sheet remained with the participants. After this, the patients who agreed to take part in the research were interviewed by the main researcher (first author) who was a male trained Medical Doctor (MD) in a quiet room at each health facility at a time convenient to the participant and the researcher. Each interview lasted for approximately 30 to 40 minutes. All interviews were recorded. The research assistant (iTaukei person) helped the researcher to translate during interviews where ever required, however, permission was first sought with the participant. Interviews were carried out until data saturation was achieved during the 1-month period of data collection.

### Data management and analysis

All interview recordings were transcribed verbatim by the principal researcher. Transcription was done on the same day of the interview. A review of transcriptions was done to correct errors and to remove references of names and places to ensure anonymity for the participants. Once the transcriptions were clarified, data analysis was carried out.

Thematic analysis was used to analyse the data in this study. Thematic analysis is a qualitative research method for identifying, analysing, organizing, describing, and reporting themes found within a data set [24, 25]. The principal researcher proof read all interview transcripts and identified similar phrases and words for which numbers were assigned. The coded data that had similar characteristics were grouped together. Once grouping of similar data was completed, descriptive themes and sub themes were identified to reflect the perceptions of participants [25]. The themes and sub-themes were checked by the principal supervisor as well.

### Study rigor

Four criteria were identified that contributed to study rigor. These criteria consisted of credibility, transferability, dependability, and confirmability. Some ways to make the study more rigor included: a short introduction of the study provided verbally by the researcher to all SOPD patients on a weekly basis for the one month period, flyers that contained information about the research placed at each SOPD clinics 2 weeks prior to data collection; in-depth interviews were conducted over the period of 1 month and each interview lasted for at least 30 minutes, all interviews were recorded, principal supervisor checked each step of the research, review of transcriptions were done to correct errors by participants, purposive sampling technique used and in-depth interviews carried out until data saturation was achieved.

### Ethical considerations

Ethical approval was received from Fiji National University’s (FNU) College Health Research Ethics Committee (CHREC) and Fiji National Research Ethics Review Committee (FNRERC) and facility use approval was received from the Sub-Divisional Medical Officer (SDMO) of Lautoka and the Western Divisional Medical Officer (DMO-West). Written informed consent was taken from SOPD patients (by either signature or left thumbprint) and assurance of confidentiality and anonymity was provided to them throughout the course of the study and afterwards as well.

## Results

### Characteristics of the participants

Twenty eight SOPD patients participated in the face to face interview. There were more female participants (53.6%) compared to male participants (46.4%). Majority of the participants were from the 61 years to 70 years age group (28.6%). In terms of ethnicity, the participants were either iTaukei (35.7%) or Fijian of Indian descent (64.3%). Looking at education level, majority of the participants had studied up to secondary school level (50%) (Table 1). Each participant was assigned a number from Participant 1 to Participant 28. The gender and ethnicity of the participants is also indicated in the quotation references.

**Table 1:** Characteristics of SOPD patient participants (n = 28).

Characteristics		Frequency	Percentage
Gender	Male	13	46.4
	Female	14	53.6
Age Groups	30–40 years	7	25
	41–50	4	14.3
	51–60	5	17.8
	61–70	8	28.6
	70 and above	4	14.3
Ethnicity	iTaukei	10	35.7
	Fijian of Indian Descent	18	64.3
Education Level	No formal education	1	3.6
	Primary School Education	6	21.4
	Secondary School Education	14	50
	Tertiary School Education	7	25
SOPD Conditions	Diabetes	6	21.4
	Hypertension	7	25
	Dyslipidaemia	5	17.9
	Both diabetes and hypertension	10	35.7

### Themes and subthemes

From the thematic analysis, four major themes emerged, this included: cancer knowledge, diagnosis of cancer in a close friend/ family, barriers of communication and optimising cancer awareness. Under these major themes, sub-themes were identified, as summarized in Table 2.

**Table 2:** Themes and sub-themes of in-depth interview analysis.

Themes	Sub-Themes
Cancer knowledge	Common cancers in Fiji
	Measure of awareness
	Cancer aetiology
	Concept of cancer prevention
	Screening modalities
	Screening facilities
Diagnosis of cancer in a close friend/ family	Cancer in relations
	Cancer impact
Barriers of communication	Influences to cancer screening
	Hinderance to cancer education
Optimising cancer awareness	Strategies to improve cancer awareness
	Cancer Hub and opportunistic screening

### Theme 1: Cancer knowledge

The first few questions asked to the SOPD patients were on cancer knowledge. The patients have highlighted various aspects of cancer information that they are aware of such as common cancers in Fiji, measure of awareness, cancer aetiology, concept of cancer prevention, screening modalities and screening facilities. These various aspects are highlighted in the sub-themes below.

### Common cancers in Fiji

Participants were aware of the common cancers in Fiji but did not have much knowledge in detail about these common cancers.

“*I am only aware of cancers which women mostly get like breast cancer*, *for the males they get it as well but I am not quite sure of it*.*”* (P11, a 79-year-old FID)

Patients had some knowledge about breast cancer.

“*I know breast cancer is very common*. *But I am not sure how it starts and what it actually looks like*.*”* (P3, a 59-year-old iT)

Patients had very less knowledge about cervical cancer.

“*There is a cancer which is in the baby bag which is called cervical cancer*, *I think*. *I have heard about it*.*”* (P13, a 45-year-old FID)

### Measure of awareness

Majority of the participants had no information about cancers. Whereas some had minimal information about cancers.

“*No one usually talks about cancer so I don’t know anything about it*. *I think when the blood gets dirty then people get cancer*.*”* (P22, a 72-year-old iT)

Some patients had minimal information about cancers.

“*I have little bit of information about it but not the actual details about it because I know there are lots of versions of cancer that you can have*.*”* (P13, a 45-year-old FID)

### Cancer aetiology

Some SOPD patients believed that a person can get cancer if its genetically linked.

“*I know a family who had cancer and all her daughters also had breast cancer*. *I think cancer is linked to family*. *Cancer is inherited*.*”* (P5, a 70-year-old IT)

Few patients stated that:

“*Cancer usually runs in the family*. *If parents have cancer*, *then their children will also have cancer*.*”* (P6, a 52-year-old FID)

Participants also stated that cancer can be caused by unhealthy eating habits.

“*Cancer is caused by what you eat*. *If you eat lot of meat and less vegetables or more snacks then you can have cancer*.*”* (P11, a 79-year-old FID)

Few participants stated that individuals who are obese can develop cancer easily.

“*Some people who are fat develop cancer easily so we need to exercise to avoid getting cancer*.*”* (P28, a 35-year-old FID)

Majority of the participants stated that smoking can cause cancer.

“*When people smoke*, *they can get cancer and even if you breathe in the smoke*, *you can get cancer very easily*.*”* (P22, a 72-year-old iT)

Few female SOPD patients believed that an injury to the breasts can cause cancer.

“*I heard that when girls get punched on the breast at a young age*, *they get breast cancer*.*”* (P9, a 74-year-old FID)

One patient stated that breastfeeding babies can increase risk of breast cancer.

“*I have heard that when babies suckle on the breast too much then that can cause breast cancer and when babies head hit on the breast*, *that can cause cancer too*.*”* (P13, a 45-year-old FID)

One participant also stated that the cause of cancer is electrical wires.

“*With my family member*, *they think it’s the electric wires that gave them breast cancer*. *The whole family didn’t get it but just these 2 sisters*. *The sisters were staying in a cottage where there were lot of electrical wires and telecom wires and they used to play around that area so we all think they must have got the cancer from there*.*”* (P1, a 63-year-old iT)

### Concept of cancer prevention

Some participants stated that cancer cannot be prevented.

“*Once you get cancer you die*. *There is no way to get saved from cancer*.*”* (P6, a 52-year-old FID)

Cancer can be prevented by practicing a healthy proper diet.

“*Balanced diet and more green vegetables can prevent you from getting cancer*. *After all*, *your body is what you eat so if you eat right*, *you will not get any disease*.*”* (P9, a 74-year-old FID)

Few of the patients were supportive of testing early to prevent getting cancer.

“*I think we should get tested for cancer*. *If its early then it can be prevented and we will not get the severe form of cancer*.*”* (P8, a 48-year-old iT)

### Screening modalities

Majority of the patients were unaware about screening modalities.

“*I don’t know how they test for cancer*. *I just know that they do some blood test for cancer*.*”* (P10, a 64-year-old iT)

Few patients knew about pap smears.

“*I think the doctors do a pap smear test to check for cervical cancer*. *It is very simple actually and doesn’t take too long*.*”* (P15, a 36-year-old FID)

Few patients also stated about mammography as a form of cancer screening tool.

“*One of my friends had a breast scan to check for breast cancer*.*”* (P14, a 31-year-old FID)

### Screening facilities

Majority of the patients stated that they are unaware where cancer screening is being conducted.

“*Honestly speaking*, *I don’t know where they test for cancer*. *That is why I have never got a cancer test done*.*”* (P19, a 57-year-old FID)

The participants have highlighted that they don’t know whom to ask for assistance regarding cancer screening.

“*I don’t know whom to ask if we want to have a cancer test done*. *Sometimes we ask the nurse but the nurse doesn’t know herself*.*”* (P21, a 67-year-old FID)

### Theme 2: Diagnosis of cancer in a close friend/family

Further questions were asked to find out about cancer amongst participants’ family members and how it has impacted them. Cancer in relations and its impact was highlighted.

### Cancer in relations

Many participants stated that they have a close family or a close friend or someone in the community diagnosed with cancer.

“*My husband has prostate cancer*. *It’s a very sad disease*. *It usually caused death*.*”* (P11, a 79-year-old FID)

One patient stated that one of his friends had cancer.

“*One of my close friends had cancer*. *He couldn’t survive*.*”* (P25, a 37-year-old iT)

### Cancer impact

Some participants highlighted that cancer in a family member did not have any effect on them.

“*Cancer in my family didn’t affect me*. *I carried on with my normal routine*. *I knew I didn’t have cancer*.*”* (P9, a 74-year-old FID)

Some participants stated that cancer in the family has significantly affected them and prompted them to get screened.

“*One of my family members had cancer and when she died*, *we were all sad and decided that we should all get checked for cancer because the doctors told us that if my family member had come in earlier*, *she could have been fully treated*.*”* (P12, a 45-year-old iT)

### Theme 3: Barriers of communication

Few questions were asked about barriers to cancer screening and cancer education. The patients highlighted many barriers. Influences to cancer screening is presented first followed by hinderance to cancer education

### Influences to cancer screening

There are many reasons why people don’t present to health facilities to get screened. Majority of the patients stated that stigmatization is a big influence on cancer screening.

“*I once went for pap smear and when I came back*, *the village ladies started talking that I had cancer and they started giving me all the advices on how to get saved from cancer*.*”* (P25, a 37-year-old iT)

Opinions of others also has a big impact on cancer screening.

“*Whenever I want to go and get tested for cancer*, *I always think that what will others think about me*. *I don’t want them to think I have cancer*.*”* (P28, a 35-year-old FID)

Some patients reported that cancer screening is a frightening experience.

“*I heard cancer test is very scary and painful*. *One of my family members went to get a cancer test done and she said it was very scary*.*”* (P4, a 79-year-old iT)

Some patients stated that they lack information about cancers hence they haven’t got themselves tested yet.

“*We don’t know much about cancer so when I don’t know about something*, *I will not get it done or do it*. *That is why I didn’t get my cancer test done because I don’t know much about cancers*.*”* (P16, a 57-year-old FID)

Trust in herbal medicine has been indicated as a barrier for screening.

“*Herbal medicine is good*. *One of the old ladies in my village knows about all the herbal medicine for all disease so if someday I have cancer*, *I will get checked by her first*.*”* (P18, a 63-year-old iT)

Lack of resources at the health facility as a barrier to cancer screening was indicated by the participants.

“*I don’t go to the health centre to get checked because majority of the time they don’t have the equipment or the bottle to test for cancer*. *One of my family members went to get checked for cervical cancer and the doctor said they don’t have the pap smear test bottle*.*”* (P22, a 72-year-old iT)

### Hinderance to cancer education

There are many difficulties faced by patients in order to access quality information about cancer. Majority of the participants attributed their poor cancer knowledge on the lack of awareness.

“*We are not aware about cancer so that’s why we don’t have much knowledge about cancer*. *If someone tells us about cancer then we will know about it*.*”* (P18, a 63-year-old FID)

Some patients have stated that cancer is a less talked about topic.

“*When we sit in family functions or in gatherings*, *no one talks about cancer even though they talk about someone having cancer but no one talks about how they got cancer*. *Usually people hide cancer as in some cultures cancer means death*.*”* (P1, a 63-year-old iT)

Another important barrier to cancer education is language barriers.

“*The advertisements and charts in health centres use lot of medical words and they speak in English so it’s a bit difficult for us to understand*.*”* (P22, a 72-year-old iT)

### Theme 4: Optimising cancer awareness

Cancer awareness is the public health intervention which is needed to combat the growing issue of cancer. Under this theme, strategies to improve cancer awareness, cancer hub and opportunistic screening are highlighted.

### Strategies to improve cancer awareness

Majority of the participants have expressed that community visits and household visits are the best was to create awareness about cancers.

“*I think when nurses and doctors come to our village and explain to us about cancer then we understand it much better*. *Usually*, *it’s good if they come because when we go to health centres*, *the doctors and nurses are very busy so they don’t explain well*.*”* (P19, a 57-year-old FID)

Few patients stated that face to face discussion while conducting house visits is an effective way to improve cancer awareness.

“*House to house visit is a very good way to explain about cancer and also to test for cancer at the same time*. *Some of us might not be confident to talk about cancer in public and when medical staffs come to our homes*, *we can explain and understand better*.*”* (P22, a 72-year-old iT)

Few participants have highlighted that cancer survivors are one of the best people to advocate about cancer to the general public.

“*When people talk with experience it makes everyone understand better*. *Therefore*, *if someone with cancer or someone who has been treated for cancer explains about cancer to the general public*, *it will be easier to understand*.*”* (P27, a 31-year-old FID)

### Cancer Hub and opportunistic screening

The concept of a cancer hub was discussed by few participants.

“*I don’t want to go to a health centre and wait for long to get tested for cancer*. *It would be good if we just go to one place like one health facility in Lautoka and get checked quickly and later follow up results from the same place and get treatment at the same place as well*. *Many people will go to this one place for cancer related issues like one stop shop*.*”* (P23, a 53-year-old FID)

Few patients stated that cancer screening should be available in all health centres routinely.

“*I think cancer testing should be done in all health centres*. *When we come to the health centre to get checked for a disease*, *the same time we can get checked for cancer too*. *This saves us time and money to go here and there to get tested for cancer*.*”* (P7, a 65-year-old FID)

## Discussion

### Cancer knowledge

The increase in cancer incidence and mortality is more prevalent in developing countries compared to developed countries [26]. This can be attributed to early detection which is easily achieved in developed countries with more facilities and awareness. However, in developing countries, due to low cancer knowledge and awareness, cancer is usually detected in later stages which contributes to the higher mortality rates [27]. Literature published highlighted that the top 5 cancers in Fiji are breast cancer, prostate cancer, cervical cancer, colorectal cancer, and liver cancer [28]. In this study, it was found that the level of awareness about common cancers in Fiji was low among SOPD patients.

Majority of the participants stated that they were aware about breast and cervical cancer since there is a lot of awareness regarding these two cancers in the media. Findings from a study by Samat et al., (2014) revealed that 96% of the participants in the study have heard of cancer and have some knowledge about it while 4% of the participants have never heard of cancer [29]. Similar findings were illustrated by Okabia et al., (2006) [30].

The SOPD patients reported lifestyle risk factors as a major contributor for cancer development. This finding is consistent with the literature whereby it was found that getting older, poor diet, smoking, alcohol, obesity are important risk factors for cancer development [31–33]. Another study showed that 67.6% of participants have knowledge of cancer and cancer risk factors, while 32.4% have no clue about cancer risk factors. Cancer risk factors stated by participants are categorized into four namely: food, family history or genetics, lifestyle, and the environment as cancer risk factors [34].

Danaei et al., (2005) highlighted that smoking, alcohol use, obesity and unhealthy diet are common risk factors for cancer development which is consistent with the finding from this study [31, 35]. Majority of the participants stated that unhealthy diet and obesity are two of the most important risk factors for cancer. This finding could be attributed to the fact that SOPD patients are regular visitors to health centres and they are often told that diet is a major risk factor for all diseases hence they strongly belief that poor diet is linked to cancer development.

Few of the patients stated that increase in meat intake and increase in oily foods can cause cancer. Fiji is a developing country which has transformed from people eating fresh farm produce to people eating processed tinned food and meat. This transformation has also noted a greater increase in lifestyle related diseases which is why the participants believed that high meat intake and oily food is a risk factor for cancer.

In this study, it was also highlighted by participants that high levels of alcohol intake and smoking posed a higher risk for cancer development. This finding is consistent with literature. Morch et. al., (2007) supported the idea that high levels of consumption of alcoholic beverages such as beer and liquors is associated with increased risk of cancer development [36]. Active exposure to cigarette smoking is a risk factor for colorectal cancer which was found by Botteri et al., (2008) [37].

One participant in this study stated that stress is a risk factor for cancer which is similar to the findings from Song et al., (2017) [38]. The stress level is further attributed to the type of work done by the individual or the environment in which the participant lives. However, there were contrasting studies which noted that there is no independent link between cancer development and stress [39].

Few SOPD patients believed that genetics and having a strong family history is a risk factor for cancer. This finding is similar to a study whereby the author found that 14.6% and 4.5% of respondents stated that family history (genetics) and the environment such as toxic pollution, respectively, to be cancer risk factors [29]. This finding can be attributed to low levels of understanding about the importance of family history. Several literatures have highlighted that family history of cancer is strongly associated with cancer development [40–42].

Few patients reported that injury to the breasts can cause cancer. This is consistent with the finding in literature whereby Peretti-Watei et al., (2014) reported that 49% of the participants said a blow to the breast is a risk factor for cancer [43]. This finding about injury to breast as a risk factor was mostly expressed by women who were told by village elders and it’s a myth that is present in villages.

### Diagnosis of cancer in a close friend/family

It was noteworthy that majority of the patients in this study stated that they had a close friend or family who was diagnosed with cancer. However, very few participants stated that this diagnosis prompted them to get screened for cancer. This finding is different from Lagarde et al., (2018) whereby they found that participants’ whose sisters were diagnosed with breast cancer served as a motivation for them to perform breast self-examination and later screening mammography [44]. Participants also valued that death is inevitable in cancer hence the need to get screened is not of concern. Another reason for this finding is that cancer prevention is not well discussed among the general public while cancer treatment is expensive in a developing country like Fiji hence the need to get screened for cancer is of no value.

### Barriers of communication

Preventive measures and effective treatment can only be achieved with early detection and appropriate treatments. SOPD patients in this study stated that they were unaware about cancer screening facilities around them and they had minimal information on how cancer screening is being done. This finding was inconsistent with the finding from the literatures as majority studies stated that participants didn’t access screening facilities due to other barriers and not exclusively because the facilities were not available or they didn’t know where to go. However, literature highlighted that problems with accessibility of services created barriers to screening. Factors such as high cost, too busy and inadequate distribution of clinics were cited as barriers to breast cancer early detection [45, 46].

Participants from a study conducted by Tatari et al., (2020) believed that cancer screening was only required if women had symptoms. This is consistent with the finding in this study as many participants stated that they had minimal information on how cancer screening is being conducted and why it is being conducted. Past studies have also similar findings that lack of knowledge of breast cancer as the factor that inhibited mammographic screening [47–49]. Participants stated that cancer was a less talked about topic and there was misinformation present among public about cancer. This finding is similar to multiple studies [50–52]. The reason for not performing BSE was declared as “Do not know how to perform” [53]. This poor knowledge about cancer screening can be attributed to poor cancer awareness among the general public.

Participants during the in-depth interview in this study highlighted that cancer information and cancer screening is not easily accessible in Lautoka. One of the most important barriers is stigmatization or opinions of other people. This finding was not consistent with literature. Since Fiji is a developing country and communities are very naïve, individual’s behaviour is usually prompted by other individual’s actions. However, one study highlighted that a significant proportion of women in the study reported that they were afraid of discovering that they had cancer and there was associated embarrassment by practicing cancer screening [47].

Worry, fear and death were important barriers highlighted in this study. Some participants stated that cancer screening is a frightening experience hence they haven’t had a cancer test yet. The fear of discovering cancer and fear of the screening procedure were among the most commonly reported personal/cultural barriers to using screening services [54]. Majority of the women thought of death and suffering when asked about cancer [55]. A belief that “it is better not to know” has been reported as a barrier to screening in studies of Hispanic women [46].

Herbal medicine, cultural beliefs and myths remain the top barriers to cancer screening. Participants stated that the village setting and traditional atmosphere in Fiji promotes herbal medicine. Religious and cultural obligations of modesty, gender of healthcare providers, fear of hospitals and need for spousal approval were mentioned by participants as barriers to uptake of screening [56].

Other important barriers stated by participants was lack of resources and delay in diagnosis. Participants felt that the waiting time in hospitals was very long and sometimes this discouraged them to come to hospital for any illness. In similarity, it was found in literature that barriers to attending screening include negative experiences associated with previous mammograms, lack of physician referral, limited access to routine health care services, low perceived susceptibility to breast cancer, failure to find a mammogram reassuring, cost, and logistic challenges [57]. Fiji being a developing country is faced with many challenges to secure appropriate healthcare resources for its population. However, with the less human resources, less equipment and less resources, the health system in Fiji is doing its best to combat serious diseases.

Language barrier was highlighted by few participants. Fiji being a multiracial country, many languages are spoken hence for people to understand a concept better, it is ideal if the informant speaks the same language. A study conducted by Persky. S, (2013) stated that participants who interacted with a racially discordant virtual doctor gave less accurate risk perception at the post-test than those who interacted with a concordant virtual doctor which may be due to language barriers [58].

### Optimizing cancer awareness

There were multiple views regarding cancer prevention. Some SOPD patients believed that cancer cannot be prevented. The views about cancer prevention are usually linked with the amount of information the individual has about cancer. This finding is similar to Meerith et al., (2012) who found in a study that participants with high cancer risk perception, were found to have a higher prevalence of good preventative behaviours [59]. Participants in this study outlined various ways to prevent cancer such as balanced diet, avoiding stress, avoiding alcohol, and avoiding smoking which is similar to literature [60].

One participant believed that breasts should be saved from injuries to prevent getting breast cancer. One literature stated that some participants mistakenly believed that regular breast massage, drinking soy milk or vaccine can prevent breast cancer [61]. Literature based evidence suggests that early cancer screening can reduce cancer mortality and prevent cancer, however only a few participants in this study believed that early cancer testing is beneficial [62, 63]. This finding can be attributed to the fact that low level of cancer knowledge existed in the population and cancer is looked as a deadly disease.

Majority of the participants stated that community outreach programs are beneficial to improve awareness. Literature highlighted similar views that cancer awareness can be done by providing screening facilities in strategic locations, especially in rural areas and setting up health campaigns to educate and provide early exposure of cancer to everybody [29, 64]. The participants stated that cancer discussion with communities followed by house-to-house visits for cancer screening would be the most beneficial way to combat the growing issue of cancer. In the interior villages of Fiji, usually the people are shy or are not confident to talk about cancer in public, hence house to house visits could be very beneficial.

The participants also stated that community health workers and cancer survivors also play a vital role in community awareness. Public health interventions need to be culturally tailored knowing that Fiji is rich in its culture and tradition. Another important concept of a cancer hub formulation whereby all cancer screening, diagnosis and treatment would be done under one roof was brought up by participants however the negative side of this project could be a risk of stigmatization. An alternative to cancer hub is incorporating cancer screening into GOPD services at health facilities to reduce stigma.

### Limitations

Findings of this research must be interpreted within the context of its limitations. These limitations include due to cross sectional design, study is limited to SOPD patients in Lautoka and findings may not be generalized to Fiji’s population. Though data saturation was achieved while conducting in-depth interviews, the sample size was very small. Another limitation found was that this study was conducted only in health facilities in Lautoka which are mostly situated in urban areas hence leaving out the health facilities which are situated in rural areas who might have different perceptions about cancer.

## Conclusions

In conclusion, SOPD patients had less knowledge about cancer risk factors and common cancers in Fiji. This has been attributed to the presence of many barriers such as stigmatization, lack of resources, poor accessibility to screening facilities, cultural beliefs, myths, fear, worry, and lack of accurate information. Community outreach programs, house to house visits and specialized cancer screening centres were recommendations given to increase cancer awareness among the general public. It is evident that there is a range of views from patients towards cancer and it is very important to understand these perceptions to better guide public health interventions concerning cancer. This puts more focus on the need to invest more in information, education and communication materials for public campaigns that target a variety of people for a wider reach.

## Supporting information

S1 Checklist (DOC)
